# DEAD-Box Helicase DDX6 Facilitated RIG-I-Mediated Type-I Interferon Response to EV71 Infection

**DOI:** 10.3389/fcimb.2021.725392

**Published:** 2021-08-13

**Authors:** Rui Zhang, Min Cheng, Bingxin Liu, Meng Yuan, Deyan Chen, Yujiong Wang, Zhiwei Wu

**Affiliations:** ^1^ Center for Public Health Research, Medical School, Nanjing University, Nanjing, China; ^2^ School of Life Sciences, Ningxia University, Yinchuan, China; ^3^ Medical School and Jiangsu Key Laboratory of Molecular Medicine, Nanjing University, Nanjing, China; ^4^ State Key Lab of Analytical Chemistry for Life Science, Nanjing University, Nanjing, China

**Keywords:** EV71, DDX6, RIG-I, Innate immune response, 2A^pro^

## Abstract

Previous studies have shown that DEAD (Asp-Glu-Ala-Asp)-box RNA helicases play important roles in viral infection, either as cytosolic sensors of pathogenic molecules or as essential host factors against viral infection. In the current study, we found that DDX6, an RNA helicase belonging to the DEAD-box family of helicase, exhibited anti-Enterovirus 71 activity through augmenting RIG-I-mediated type-I IFN response. Moreover, DDX6 binds viral RNA to form an RNA-protein complex to positively regulate the RIG-I-mediated interferon response; however, EV71 has evolved a strategy to antagonize the antiviral effect of DDX6 by proteolytic degradation of the molecule through its non-structural protein 2A, a virus-encoded protease.

## Introduction

Enterovirus 71 (EV71) is a positive-stranded RNA virus that contains a single open reading frame and is non-encapsulated (Wang and Li, 2019). Since its outbreak, EV71 has caused serious pandemics in Asia, including hand-foot and mouth disease (HFMD), encephalitis, and flaccid paralysis (Weng et al., 2010).

Interferons are important to antiviral therapy as the first line of the host immune response to viruses. Among these, the host senses pathogenic microbial invasion by recognizing pathogenic molecules using recognition receptors, including the classical Toll-like receptor (Meylan and Tschopp, 2006; Takeda and Akira, 2015), the cytosolic sensor RIG-I and MDA5. Many viruses of the picornavirus family, such as enteroviruses, rhinoviruses polioviruses, have evolved multiple strategies to antagonize the interferon response by disrupting the pathogen-sensing receptors MDA5 and RIG-I (Barral et al., 2007; Barral et al., 2009). Particularly, the RIG-I signaling pathway is complexly regulated at multiple levels. Y. Hu et al. have reported that the ubiquitination of RIG-I is regulated by the coronavirus N protein (Hu et al., 2017). Bunyavirus-encoded NSs specifically inhibit the ubiquitination/activation of RIG-I, thereby suppressing RLR-mediated antiviral signaling (Min et al., 2020). Because of the important role of RIG-I signaling in the antiviral immune responses, it is crucial to investigate the function of proteins regulating RIG-I.

DEAD-box RNA helicases (DDX) family has been shown to participate in every aspect of RNA metabolism, including pre-mRNA processing, translation, RNA decay, ribosome biogenesis, transcription, and gene expression regulation in RNA viruses (Rocak and Linder, 2004; Presnyak and Coller, 2013). It is supported that DDX6 has pro- and antiviral roles in viral infections. DDX6 modulates replication and the stability of HCV RNA through interaction with miR-122 and the viral-5′UTR (Biegel et al., 2017). Bunyaviruses snatch mRNA caps from P-body component DDX6, promoting the decapping of host mRNAs and decreasing the capped RNAs in P bodies to reduce virus infection (Hopkins et al., 2013). In mosquitoes, DDX6 plays a role against the flavivirus, such as West Nile virus and Zika virus. In a recent report, DDX6 is counteracted by the noncoding sfRNA derived from the flavivirus 3’ UTR, which binds to and sequesters DDX6 (Goertz et al., 2019).

Additionally, viruses could hijack P-body components to aid their replication. Ward et al. have demonstrated that DDX6 and other SG components bind dengue virus 3’ UTR RNA to facilitate virus replication (Ward et al., 2011). However, the role of DDX6 remains unclear during EV71 infection. Given the important roles of DDX6 in viral infection, we investigated the role of DDX6 in EV71 infection.

## Experimental Procedures

### Cell Lines and Viruses

HeLa, HEK-293T, Vero, and RD cells were obtained from ATCC and cultured in DMEM (Gibco, USA) containing 10% fetal bovine serum in a 5% CO_2_ and 37°C humidified atmosphere. Enterovirus 71(FY0805 strain) was a kind gift from Prof. Erguang Li (Nanjing University). Detailed protocols for viral amplification and titer were carried out according to our previous report (Lv et al., 2014).

### Western Blot

Western blot in this study was performed according to standard procedures as our previous description (Fu et al., 2019). The primary antibodies were used in this study as followed: anti-VP1 (MAB979, Millipore), anti-DDX6 (A300-460A, Belthy-Lab), anti-GAPDH (60004-1-Ig, Proteintech), anti-RIG-I (#3743), MAVS (#3993), eIF4G (#2469), IRF3 (#11904) and P-IRF3 (phosphor-S386) (#29047) were from Cell Signaling Technology.

### Co-Immunoprecipitation Analysis (Co-IP)

HEK-293T cells were transfected with the vectors encoding Flag-RIG-I and GFP-NC or GFP-DDX6 through Lipofectamine 3000 (Life Technologies, USA). After 36 h transfection, cells were lysed with RIPA buffer(Santa Cruz, USA), and the whole-cell lysate was incubated with anti-Flag (Sigma) antibody at 4°C overnight following by the Protein A+G agarose beads (Millipore, USA) incubation for additional 60 minutes. After washes, the beads were lysed with RIPA buffer and analyzed by Western blot with antibodies to RIG-I (rabbit, Cell Signaling Technology), GFP (mouse, Beyotime Biotechnology), and GAPDH (mouse, Proteintech). For the purification of DDX6-associated RNA, Similarly, RIP experiments were performed by incubating with DDX6 antibody. After three times washing, the immunoprecipitated protein-RNA complex was carried for RNA analysis.

### RNA Extraction and Quantitative Polymerase Chain Reaction (qPCR)

Total RNA samples were isolated using TRIzol reagent (Life Technologies) and reverse-transcribed using PrimeScript RT Master Mix (TaKaRa) for RT-PCR. Quantitative real time-PCR (qRT-PCR) was completed on ABI 7500 Sequence Detection System using SYBR Green Master Mix (Life Technologies). GAPDH gene was used for the standard of mRNA. All reactions were performed in triplicate, and data were analyzed using the 2^-ΔΔCt^ method (Fu et al., 2017). The sequences of real-time PCR primer pairs were shown in **Table 1**.

**Table 1:** The sequences of real-time PCR primer pairs.

Primer name	Sequence (5’ to 3’)
GAPDH qF	TGCACCACCAACTGCTTAGC
GAPDH qR	GGCATGGACTGTGGTCATGAG
DDX6 qF	ATGGGTCTGTCCAGTCAAAATG
DDX6 qR	GGTGGTCATACTCTGTGCTTG
IFN-β qF	ATGACCAACAAGTGTCTCCTCC
IFN-β qR	GGAATCCAAGCAAGTTGTAGCTC
IFI-27 qF	TGCTCTCACCTCATCAGCAGT
IFI-27 qR	CACAACTCCTCCAATCACAACT
OAS-1 qF	TGTCCAAGGTGGTAAAGGGTG
OAS-1 qR	CCGGCGATTTAACTGATCCTG
ISG-15 qF	CGCAGATCACCCAGAAGATCG
ISG-15 qR	TTCGTCGCATTTGTCCACCA
EV71-VP1 qF	GCTCTATAGGAGATAGTGTGAGTAGGG
EV71-VP1 qR	ATGACTGCTCACCTGCGTGTT

### Northern Blot

Using PrimeScript RT Master Mix for RT-PCR (TaKaRa), reverse transcription was achieved under standard procedures. RT products were amplified using DreamTaq Green PCR Master Mix (Tsingke Biotechnology) and amplify mRNA using the primer listed in **Table 2**. The PCR products were performed on 1.2% agarose/TAE gels containing gel red.

**Table 2:** The sequences of PCR primer pairs.

Primer name	Sequence (5’ to 3’)
EV71 RNA-Forward	GGGGTACCAGTGATATCCTGCAGACGGG
EV71 RNA-Reverse	GAAGATCTATAGCCCCAGACTGTTGTCC
Histone-Forward	ACCCTCCTCGACTTCCACAG
Histone-Reverse	TGTAGAGCTTGATAGCTGCCA

### Immunofluorescence Analysis

Immunofluorescence was performed as previously described (Fu et al., 2017). Antibodies used for staining are as followed: anti-VP1 antibody (ab36367, Abcam), Flag (F1804, Sigma), DDX6 (A300-460A, Belthy-Lab). The secondary antibodies are: Alexa Fluor goat anti-mouse-594 IgG (h+L) (1:1000 dilution), Alexa Fluor goat anti-rabbit-594 IgG (h+L) (1:1000 dilution) and Alexa Fluor goat anti-rabbit-488 IgG (h+L) (1:1000 dilution, Life Technologies). All images were taken using the Olympus FV3000 confocal microscope (Tokyo, Japan).

### RNA Interference and DNA Transfection

HeLa cells were transfected with 2.5 µg plasmid or 5um siRNA in a 6-well plate using Lipofectamine**™** LTX Reagent and Lipofectamine RNAi Max transfection reagents (Invitrogen), respectively, according to the instructor’s manual. The siRNA scramble sequence was 5’-GCAUGAAUCGAGGCCCAAUU-3’. The siRNA specific for the Human DDX6: 5’-GCAGAAACCCUAUGAGAUUU-3’. HeLa or 293T cells were transfected with plasmids: pEGFP-C1, pEGFP-C1-DDX6, pcDNA3.1-Flag-DDX6, pcDNA3.1-Flag-RIG-I, EV71 recombinant 3C/2A/2A^mut^, IFN-β, and p125Luc using Lipofectamine**™** LTX Reagent for 24 h followed with non-infection or EV71 infection (MOI = 5) for additional 24 h. Total protein was lysed in RIPA buffer supplemented with Protease Inhibitor Cocktail (Santa Cruz, USA).

### Luciferase Reporter Assays

HEK-293T cells were transfected with recombinant expression plasmids (Vector or DDX6) and the p125-Luc (IFN-β promoter-luciferase reporter plasmid, experimental reporter). After 24h transfection, cells were treated with EV71 (MOI=2) or Poly (I: C) (500 ng), and luciferase activity was detected using a Dual Glow Kit (Promega, USA).

### TCID_50_ Assay and Viral Growth Kinetics

The cell supernatants were collected, and the titers of EV71 were determined with a TCID_50_ assay according to the standard procedure on Vero cells as described previously (Zhong et al., 2017). Quantification of intracellular viral replication levels was determined with Q-PCR, normalized to GAPDH.

### Statistical Analysis

Graphical representation and statistical analyses were performed using GraphPad Software8.0.1. Results are shown as means ± standard deviation (SD) from three independent experiments. The differences among treatment groups were analyzed by the Student’s *t-*test or One-way Analysis of Variance (ANOVA) followed by Bonferroni’s multiple comparisons test. *P-value < 0.05 was considered significant; **P < 0.01 was considered highly significant.

## Results

### DDX6 Was Down-Regulated Upon EV71 Infection

**Figure 1:**
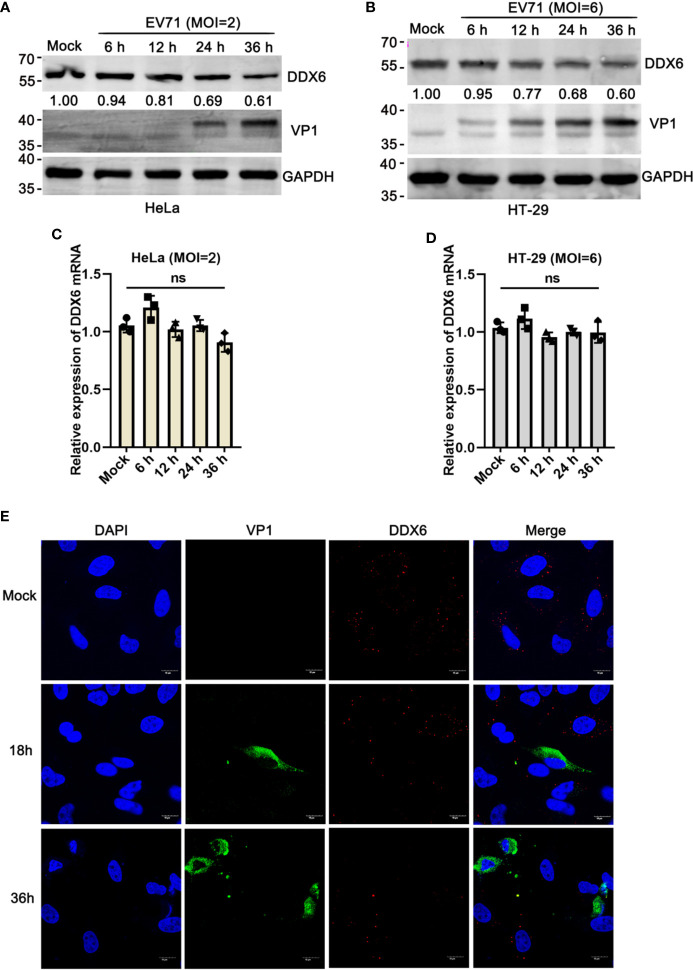
EV71 reduced P-body component DDX6 but did not affect *de novo* expression. **(A, B)** EV71 infection reduces endogenous DDX6. HeLa and HT-29 cells were infected with EV71 under different MOIs for the indicated times, respectively. Cell lysates were determined by Western blot using antibodies against DDX6, VP1, and GAPDH. The value of DDX6 in uninfected cells (Lane1) is set as 1.00. The representative data are shown. **(C, D)** The mRNA of DDX6 showed no change after EV71 infection. The total cell RNA was extracted, and DDX6 mRNA was analyzed by real-time PCR assay, GAPDH as control. All these experiments were performed three times, and representative results were shown. *ns* indicate no significant difference. **(E)** HeLa cells grown on 12-well slides were infected with EV71 (MOI=2) or control treatment. After the corresponding time treatment, the cells were fixed and stained with antibodies against DDX6 (red), VP1 (green), and DAPI (blue); the images were acquired using a confocal microscope, and a representative of three independent experiments was shown.

Earlier studies revealed that DDX6 primarily localizes in the cytoplasm in a dispersed, punctate form in both infected and uninfected cells, a structure known as a P-small body. Notably, the number and size of the P-bodies appeared to be reduced in cells infected with HCV (Ariumi et al., 2011). To determine whether DDX6 protein was affected by EV71, lysates from EV71 infected-HeLa or HT-29 cells were analyzed by western blot, respectively, and the results indicated that EV71 infection reduced DDX6 protein expression by about 40% as compared with that in the uninfected cells (**Figures 1A, B**). However, DDX6 mRNA showed no noticeable changes upon EV71 infection as determined by real-time PCR in HeLa and HT-29 cells (**Figures 1C, D**). Similarly, we carried out an immunofluorescence assay and observed that DDX6 decreased gradually with viral infection (**Figure 1E**). Altogether, the results suggest that EV71 infection impaired P-body component DDX6 at the protein but not the mRNA level.

### DDX6 Knockdown Enhanced EV71 Replication

**Figure 2:**
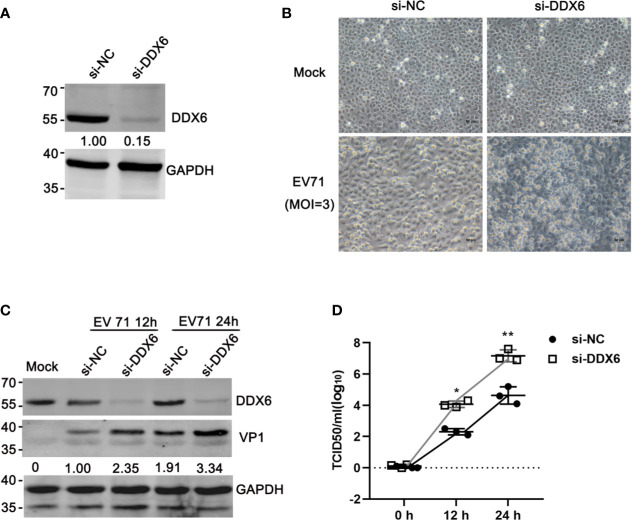
Knocking down DDX6 facilitated EV71 replication. **(A)** Efficiency of DDX6 downregulation by si-DDX6 in HeLa cells. HeLa cells were treated with either si-NC (a scrambled sequence) or si-DDX6. The total protein extract from infected cells was analyzed by Western blot using antibodies against DDX6 and GAPDH (loading control). **(B)** Cells transfected with either si-NC or si-DDX6 grown in 12-well plates were infected with EV71 (MOI =3) for 36 h. The EV71-induced CPE in the DDX6^+/+^ and DDX6^-/-^ cells were photographed using microscopy (scale bar: 50 µm). **(C)** After si-NC or si-DDX6 transfection for 36 h, the relative VP1 expression of HeLa cells 12 or 24 hpi was shown, respectively. Densitometry was performed, and lane 2 is set as 1.00. **(D)** Analysis of progeny virus in culture supernatants by a standard plaque assay. The values shown are the means ± SD of three determinations (*P < 0.05, **P < 0.01).

To investigate the roles of DDX6 during EV71 infection, endogenous DDX6 was silenced by RNAi. siRNA targeting DDX6 significantly reduced DDX6 mRNA and protein expression compared with the cells transfected with scrambled siRNA (si-NC), as shown by qPCR and western blot analysis (**Figure 2A**). As shown in **Figure 2B**, EV71 infection of the cells with endogenous DDX6 knockdown caused more cytopathic effect than the si-NC treated cells infected with EV71. To further confirm this observation, we performed western blot analysis of viral structural protein and showed that VP1 expression correspondingly increased 2~3 fold in DDX6-knockdown cells as compared with the si-NC treated cells at various times (**Figures 2C**, Lanes 3, 5, compared with Lanes 2, 4, respectively). Next, we determined the effect of DDX6 on EV71 progeny virus production by measuring the TCID_50_ of the virus collected from cultural supernatants of si-DDX6 treated Vero-E6 cells. As shown in **Figure 2D**, progeny virus in the DDX6 knockdown cells increased by 2-log 12 h post-infection (hpi) and by 3-log 24 hpi. Altogether, the data suggested that DDX6 downregulation renders more efficient replication of EV71, suggesting the important roles of DDX6 in EV71.

### DDX6 Overexpression Impaired EV71 Gene Expression

**Figure 3:**
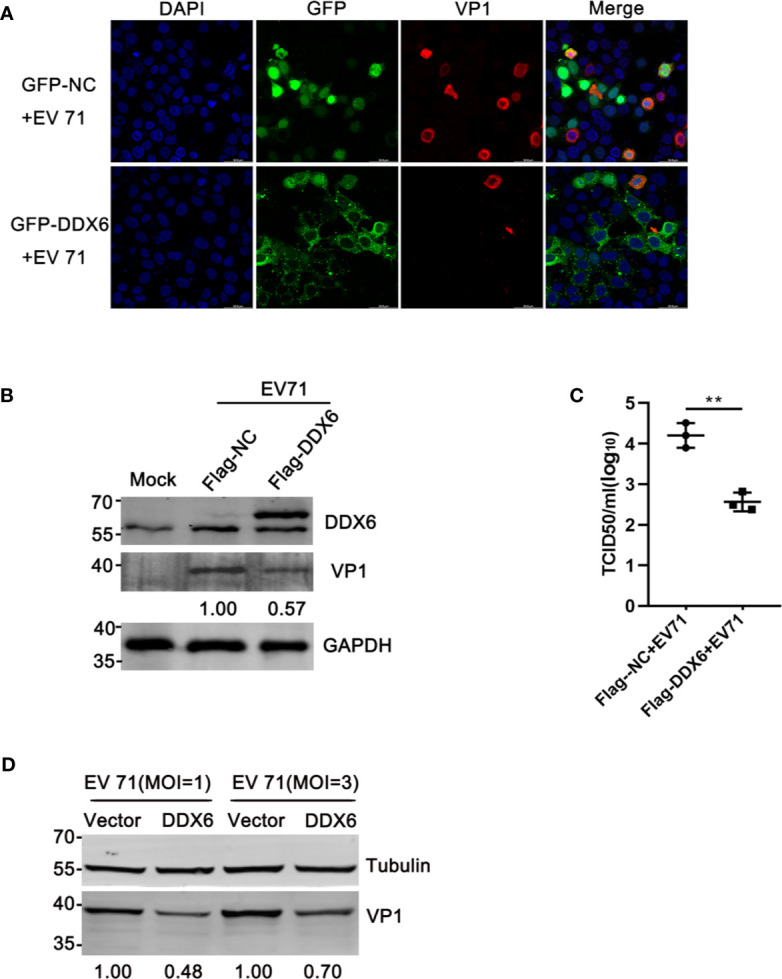
DDX6 conferred cellular resistance to EV71 infection. **(A)** The plasmids expressing GFP-NC or GFP-DDX6 were expressed in HeLa cells, and the cells were infected with EV71 (MOI=2) for 24 h. The infected HeLa were fixed and stained by staining with anti-VP1 antibody (red), and the cell nuclei stained with DAPI (blue). The images were taken with an Olympus FV3000 confocal microscope, scale bar: 50 µm. **(B)** HeLa cells were transfected with a plasmid expressing DDX6 (pcDNA3.1-Flag-DDX6) or a control plasmid (pcDNA3.1-Flag). After 24 h of transfection, the cells were treated with EV71 (MOI=2). At 24 hpi, the cell lysates were analyzed by western blot using antibodies specific for DDX6, VP1, and GAPDH. The value for lane 2 was 1.00. **(C)** The amount of progeny virus in the supernatant from treated cells was determined through a standard plaque assay. The values represented three independent experiments (*n* = 3, **P < 0.01). The error bar represented mean with SD. **(D)** The relative VP1 expression in vector or DDX6 over-expressed cells infected with EV71 (MOI = 1 or 3) was determined by Western blot.

The above observations suggest that DDX6 played a negative regulatory role during EV71 infection. To confirm this, we carried out a reverse assay by monitoring EV71 replication in cells with DDX6 overexpression. As shown in **Figure 3A**, the indirect immunofluorescence assay showed that DDX6 overexpression reduced EV71 infection (positive VP1 staining indicates the infected cells) as compared with a control vector in the HeLa cells at 24 hpi Meanwhile, Flag-DDX6 overexpression resulted in VP1 reduction by 43% compared to HeLa with a Flag-vector at 24 hpi (**Figure 3B**); the upper band represents the overexpression. Also, the same results were observed in HEK 293 cells containing lower levels of endogenous DDX6 (**Supplementary Figure 1B**). Similarly, progeny virus in the cells expressing DDX6 was lower than in the cells transfected with the vector only (**Figure 3C**). To further demonstrate the effect of DDX6 on EV71, HeLa cells were infected with various MOIs for 24 h, and VP1 expression was shown suppressed in DDX6 overexpressed cells (**Figure 3D**). These results indicate that the exogenously expressed DDX6 significantly suppressed EV71 replication.

### DDX6 Loss Weakened Type I-IFN Response to EV71 Infection

**Figure 4:**
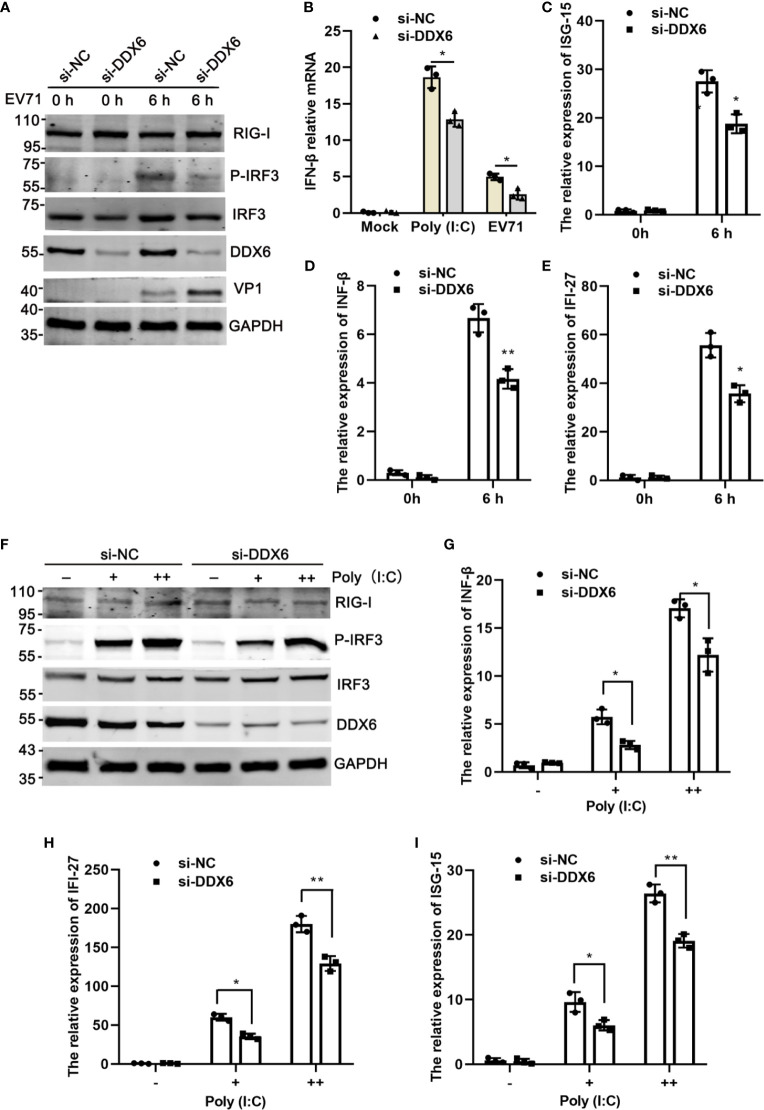
Knockdown of DDX6 reduced I-IFN activation during EV71 infection. **(A)** HeLa cells were transfected with si-NC or si-DDX6 for 48 h and then infected with EV71 6 h p.i., then was analyzed by western blot with the indicated antibodies (RIG-I, P-IRF3, IRF3, DDX6, and VP1). GAPDH was used as a control. **(B)** After Poly (I: C) and EV71 stimulation, the relative expression of exogenous IFN-β was detected by real time-PCR in si-DDX6 and si-NC transfected cells. **(C–E)** The mRNA levels of IFN-β, IFI-27, and ISG15 were analyzed by real-time PCR and normalized to GAPDH. **(F)** HeLa cells were treated with si-NC or si-DDX6 for 48 h and treated with or without poly (I:C) (+/++ represent 200 ng and 500 ng, respectively) for 12 h, western blot analysis of cell lysates with antibodies specific for RIG-I, MAVS, P-IRF3, IRF3, DDX6, and GAPDH. **(G–I)** The relative mRNA expression of IFN-β, IFI-27, and ISG15 was determined by a real-time PCR assay. The results are presented as means with SD (*P < 0.05, **P < 0.01).

Type I-IFN plays a critical role in the innate immune response as the first line of defense against enterovirus infection and regulates many immune responsive genes, including IFN-β, IFI-27, ISG15, and OAS-1 (Schoggins et al., 2011; Wu and Chen, 2014; Roers et al., 2016). The RNA helicase RIG-I has an important role in the innate antiviral response induced by viral RNA (Yoneyama et al., 2004; Platanias, 2005). Based on the above results that DDX6 played an inhibitory role during EV71 infection, we speculate that DDX6 regulates IFN response during virus infection. Upon endogenous DDX6 knockdown by si-DDX6, p-IRF3 decreased in siDDX6-treated cells compared with that in siNC-treated cells upon EV71 infection (**Figure 4A**). Consistent with the above results, qPCR assay further confirmed that ISG15, IFN-β, and IFI-27 were reduced in the siDDX6-transfected group during EV71 infection compared with those in the siNC-transfected group (**Figures 4C–E**). To further illustrate the role of DDX6 in regulating type I-IFN, poly (I:C), a strong stimulant recognized by RIG-I or MDA5 (Kato et al., 2008; Uzri and Gehrke, 2009), was used to treat the cells and poly (I:C)-induced IFN-β upregulation was inhibited when DDX6 was knocked down (**Figure 4B**). Consistently, p-IRF3 was also reduced under the stimulation of poly (I:C) in siDDX6-treated HeLa cells, as shown in **Figure 4F**. At the same time, the mRNA level of ISGs (IFN-β, IFI-27, ISG15) was reduced in the DDX6-silenced cells (**Figures 4G–I**). Altogether, these findings corroborated the observation that DDX6 downregulation weakened type I-IFN response to EV71 infection.

### DDX6 Suppressed EV71 by Augmenting RIG-I Mediated Type-I IFN

**Figure 5:**
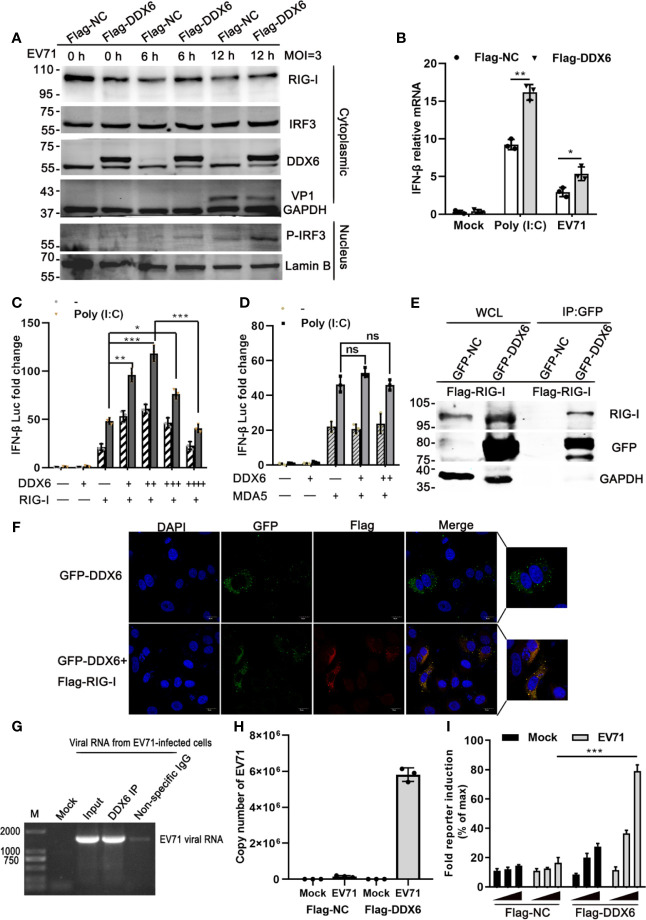
DDX6 positively regulates RIG-I and promotes the activation of type-I IFN during EV71 infection. **(A)** HeLa cells were transiently transfected with a plasmid expressing DDX6 or control plasmid (pcDNA3.1-flag). After 24 h transfection, the cells were infected with EV71 at (MOI = 3) for 6 h and 12 h, respectively. The nucleus and cytoplasm extracts were determined by western blot using antibodies specific for DDX6, VP1, RIG-I, MAVS, IRF3, P-IRF3, Lamin B, and GAPDH were used as the control for nucleus and cytoplasm, respectively. **(B)** The relative mRNA of IFN-β in HEK-293T was cotransfected with the plasmid vector or Flag-DDX6 and IFN-β for 24 h and stimulated by poly (I: C) or EV71 for 12 h, respectively. Data from three independent experiments were averaged. **(C)** Vectors expressing RIG-I, DDX6 were transfected into HEK-293T cells together with p125Luc (the IFN-β-luciferase reporter plasmid). After 24 h, the cells were unstimulated or stimulated with poly (I: C) for 12 h. Cell lysates were prepared, and luciferase activity was measured. +/++/+++ represents an increasing quantity of plasmid. **(D)** Vectors expressing MDA5, DDX6 were transfected into HEK-293T cells together with the IFN-β-luciferase reporter plasmid. After 24 h, the cells were left unstimulated or stimulated with poly (I: C) for 12 h. Cell lysates were collected, and luciferase activity was measured. **(E)** HEK-293T cells were transfected with Flag-RIG-I for 24 h, followed by transfection with plasmids encoding DDX6 or vector. Co-IP and immunoblotting were performed with the indicated antibodies. **(F)** HeLa cells were cotransfected with plasmids GFP-DDX6 and Flag-RIG-I for 36 h, fixed and stained with monoclonal antibodies against Flag (red) and DAPI (blue). Images were acquired using a confocal microscope. (scale bar: 15 µm). **(G)** For the purification of DDX6-associated RNA, HeLa cells infected with EV71 were incubated with DDX6 antibody and control IgG, and total RNA was isolated and analyzed for EV71 RNA by Northern blot. Nonspecific rabbit IgG was used as a control antibody. **(H)** HeLa cells transfected with Flag-DDX6 or Flag-NC were mock-infected or infected with EV71. Cell extracts were prepared 15 hpi, and the proteins were selected by Flag-trap, followed by purification of the associated RNAs. Subsequently, EV71 RNA copy numbers were determined by Q-PCR. **(I)** RNA eluted from Flag-DDX6, or Flag-NC precipitates as detailed in panel **(H)** was re-transfected in increasing quantity (10, 40, 90 ng) into HEK-293T cells along with Flag-RIG-I, p125-Luc, and a control *Renilla* luciferase. Cells were harvested 24 h post-transfection, and the RIG-I-mediated IFN-β activation was measured by reporter assay. Values are mean ± SD of at least three independent experiments (*P < 0.05; **P < 0.01; ***P < 0.001 *versus* control, ns indicate no significant difference).

It is known that RIG-I/MDA5 plays an important role during the picornavirus life cycle (Kato et al., 2006; Kuo et al., 2013; Lei et al., 2016). To investigate the regulatory mechanisms of DDX6 on type I-IFN response, we overexpressed DDX6 in HeLa cells for 24 h, followed by EV71 infection at various time points (0, 6, and 12 hpi) and analyzed downstream signaling pathways. As shown in **Figure 5A**, we found that exogenous DDX6 promoted P-IRF3 nuclear translocation from 12 hpi while did not alter RIG-I expression. In addition, we measured the expression levels of IFN-β under either poly (I:C) stimulation or EV71 infection and found that the presence of exogenous DDX6 facilitated a significant increase of the poly (I:C)-induced IFN-β as compared to the control group; however, interferon response was lower in EV71 infection than poly (I:C) stimulation, which is consistent with the inhibition of interferon response by EV71 infection (Lei et al., 2010; Feng et al., 2014) (**Figure 5B**). We next explored how DDX6 regulates upstream receptors to enhance interferon response. It has been reported that DDX6 acts as a positive regulator of RIG-I in the course of influenza A virus infection (Nunez et al., 2018). To explore the potential roles of DDX6 on the RIG-I signaling pathway, we cotransfected an increasing amount of DDX6 and a constant amount of RIG-I and found that DDX6 positively regulated the activation of RIG-I on IFN-β in a dose-dependent manner. IFN-β-Luc activity with poly (I:C) stimulation increased in a dose-dependent manner to increasing DDX6 and reached a plateau and then decreased as DDX6 increased further, as shown in **Figure 5C**, suggesting a negative feedback mechanism to regulate over-activation of the RIG-I-mediated IFN-β promotion. However, the IFN-β activation mediated by MDA5 was not dependent on the DDX6, as shown in **Figure 5D**, suggesting that DDX6 positively regulates the interferon response by modulating RIG-I but not MDA5. Confocal microscopic analysis showed the intracellular co-localization of RIG-I and DDX6, as shown in **Figure 5F**. In non-infected cells, both RIG-I and DDX6 were diffusely distributed throughout the cytoplasm, suggesting a partial co-localization of DDX6 and RIG-I. co-IP was performed in cells expressing Flag-RIG-I with an antibody specific for the GFP tag on DDX6 to determine whether DDX6 binds directly to RIG-I. The results revealed that Flag-RIG-I was co-precipitated with DDX6, suggesting that DDX6 is constitutively complexed with RIG-I in 293T (**Figure 5E**). In the infected cells, we detected the association of DDX6 with EV71 viral RNA using a DDX6-specific antibody in a pull-down experiment (**Figures 5G, H**). The specific binding of DDX6 to viral RNA was validated using histone mRNA, which did not bind DDX6 and served as a negative control (**Supplementary Figure 1A**). Similarly, viral RNA and DDX6 co-localization immunofluorescence was shown in **Supplementary Figure 1C**. We postulated that the DDX6-associated viral RNA would activate RIG-I and thus the downstream IFN-β response. To test this hypothesis, RNA eluted from Flag-DDX6 or Flag-NC co-precipitates (**Figure 5H**) was re-transfected into 293T cells with Flag-RIG-I p125-Luc and a control *Renilla* luciferase, and the cells were either infected with EV71 or not. RIG-I-dependent IFN-β promoter induction was evaluated. Notably, RNA derived from DDX6 but not the NC co-precipitation of the infected cells specifically stimulated RIG-I-mediated IFN-β promoter activation in a dose-dependent manner (**Figure 5I**). DDX6-associated viral RNA can stimulate RIG-I. Altogether, our data suggest that DDX6 enhancement of type I IFN response to EV71 infection was mediated through regulating the RIG-I pathway.

### Viral 2A^pro^ Induced DDX6 Degradation

**Figure 6:**
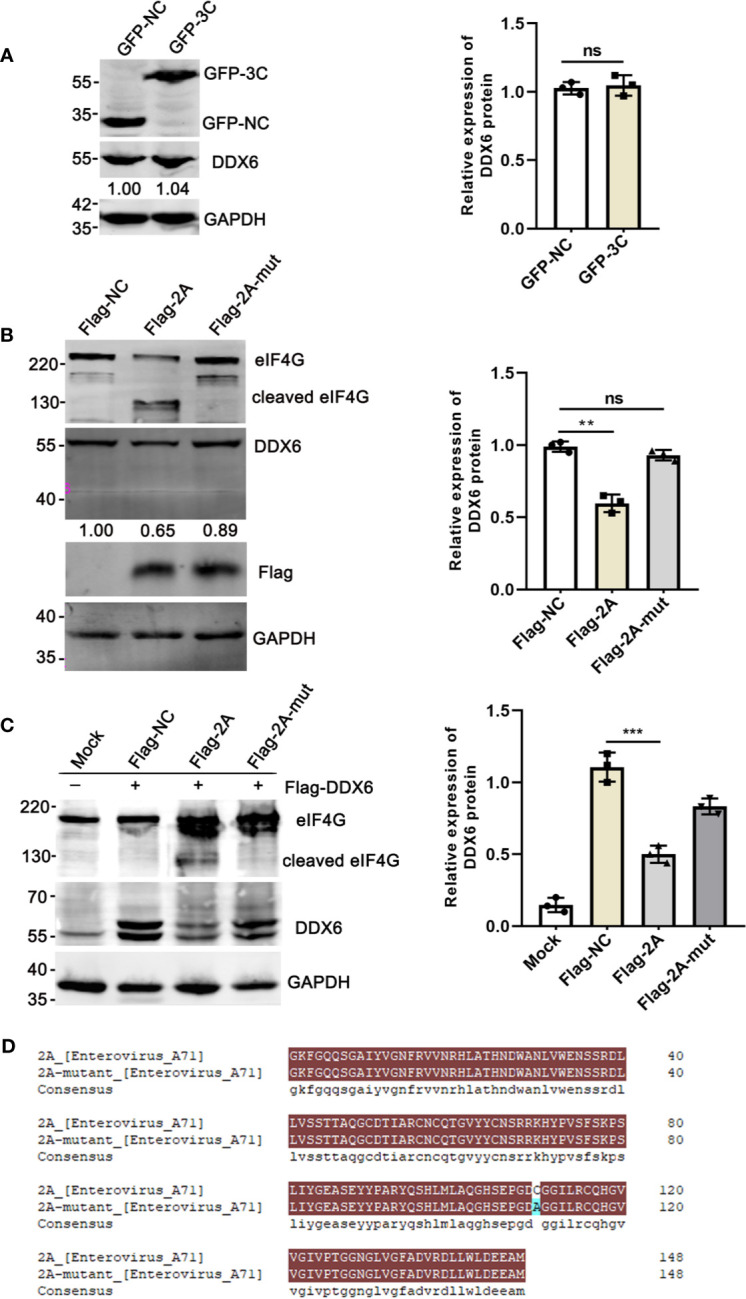
The endogenous DDX6 was degraded by viral-2A^pro^ but not 3C^pro^. **(A)** HeLa cells were transfected with an empty vector (pEGFP-C1) or plasmid encoding EV71 protease 3C for 36 h. The whole-cell lysate was analyzed by immunoblotting using specific antibodies (GAPDH, DDX6, and GFP). **(B)** HeLa cells were cultured in 6-well dishes, transfected with equal amounts of Flag-NC, Flag-2A, and Flag-2A-mutant (2500 ng), respectively. The cells were lysed and analyzed by Western blot. **(C)** 293T cells transfected with Flag-DDX6 and then transfected with equal amounts of Flag-NC, Flag-2A, or Flag-2A-mutant plasmids for 24 h, respectively. Western blot was performed using various specific antibodies (eIF4G, DDX6, and GAPDH) (**P < 0.01; ***P < 0.001, ns indicate no significant difference). **(D)** The plasmid encoding 2A-mutant was constructed by mutating the amino acid at the 110^th^ position of the wild-type 2A, and the amino acid comparison diagram of wild-type and mutant 2A was produced by DNAMAN.

Earlier studies revealed that the human P-body component Dcp1a was cleaved by the 3C protease of poliovirus (Dougherty et al., 2015). 2A^pro^ and 3C^pro^, the two viral proteases encoded by EV71, are important for processing viral protein precursors and have been reported to cleave many cellular proteins (Weng et al., 2009; Wang et al., 2013; Dougherty et al., 2015). We also showed that 3C^pro^ induced PML degradation and thus alleviated the PML-restricted autophagy upon EV71 infection (Chen et al., 2018). In addition, our observation that DDX6 was down-regulated upon EV71 infection at protein level suggests that the viral proteases may play a role. Therefore, we investigated whether the two viral proteases 2A^pro^ and 3C^pro^ could be responsible for reducing DDX6 during EV71 infection and showed that viral 3C^pro^ had no apparent impact on the endogenous DDX6 (**Figure 6A**). In contrast, DDX6 was reduced by about 35% in the presence of Flag-2A^pro^, which was not observed in the cells expressing a cleavage defective 2A^pro^-mutant, as shown in **Figure 6B**. These results suggest that 2A^pro^ caused DDX6 degradation enzymatically, which was further confirmed in 293T cells with a plasmid encoding Flag-DDX6 and plasmids encoding either Flag-2A^pro^ or 2A^pro^-mutant (**Figure 6D**), respectively. 2A^pro^ has been reported to induce stress granule formations by cleaving eIF4GI to seal cellular mRNA and release viral mRNA, facilitating viral translation (Yang et al., 2018). Dave *et al.* demonstrated that 2A^pro^ could cleave eukaryotic translation initiation factor 4G (eIF4G) (Dave et al., 2019). The exogenous DDX6 was reduced in 2A^pro^-expressing cells but not in control or 2A^pro^-mutant expressing cells (**Figure 6C**). These data showed that the viral-2A^pro^, but not 3C^pro^, specifically degraded DDX6 upon EV71 infection.

## Discussion

The current study provided evidence that DDX6, a DEAD-box RNA helicase family molecule, suppressed EV71 replication by enhancing the RIG-I signaling pathway. We found that EV71 replication substantially increased when endogenous DDX6 was knockdown in HeLa cells (**Figure 2**). DDX6 knockdown decreased the expression of interferon-stimulated factors in response to EV71 early infection (**Figure 4**). Our data indicated that DDX6 acts as a host restriction factor to inhibit EV71 while enhancing type I interferon response through regulating RIG-I. DDX6 promoted RIG-I mediated IFN-β induction in a dose-dependent manner, but high levels of exogenous DDX6 significantly lowered the activation of RIG-I mediated IFN-β promoter, indicating that DDX6 may act as a regulator of RIG-I to limit the excessive activation of cytoplasmic RNA. Previous research suggested that MDA5 is an important RNA recognition molecule in Picornaviruses infection (Feng et al., 2012), but our results suggested that DDX6 does not regulate the IFN-β induction mediated by MDA5 (**Figure 5D**). In addition, our co-immunoprecipitation identified an interaction between DDX6 and RIG-I in the presence of EV71. Of note, it has been shown that this interaction of RIG-I and DDX6 was bridged through viral RNA during IAV infection, and DDX6 enhanced the RIG-I signaling pathway by associating with RIG-I and viral RNA in infected cells (Nunez et al., 2018). Similarly, in our study, we found that DDX6-associated viral RNA could stimulate RIG-I in a dose-dependent manner (**Figure 5I**), suggesting that it is an evolutionary mechanism for cellular factors to complex with viral genomic RNA to form a stable RNP complex to stimulate RNA sensor to enhance the downstream interferon response, thus achieving the antiviral effect.

We also found that RNA bound to DDX6 slightly stimulated RIG-I-mediated activation of interferon when there was no viral infection, which may be explained by a hypothesis that DDX6 acts as an RNA-binding protein that also binds other intracellular proteins to regulate RNA sensors in the absence of viral infection. Some host factors have evolved a synergistic relationship with the innate immune molecules to fight virus infection in mammalian cells (Lu et al., 2016; Ge et al., 2017). These observations broadened the neglected cellular factor (DDX6) of regulating RIG-I activation in response to EV71 infection. However, as with many viruses, EV71 has evolved ways to overcome cellular restriction. The virus-encoded 2A protease was responsible for reducing DDX6 during EV71 infection (**Figures 6B, C**). The 2A^pro^ cleavage was specific and enzymatic, thus mitigating the capability of DDX6 in facilitating RIG-I-mediated type I IFN response. Though we failed to detect the DDX6 fragments in the infected cells, the failure of detecting cleaved fragments was either due to the DDX6-specific monoclonal antibody epitope specificity or rapid degradation of the cleaved DDX6.

DDX6 was also identified as a suppressor of aberrant ISGs activity in haploid human cells; DDX6 deletion stimulated the innate immune signaling pathway and enhanced the cellular response to IFN by disrupting cytoplasmic RNA turn over (Lumb et al., 2017). However, contrary to the previous study, the current report reveals the functional diversity of DDX6 in different cellular environments. The suppressive regulatory roles of high-level exogenous DDX6 are intriguing and suggest that DDX6 might act as a buffer to support IFN induction through association with RIG-I while limiting the adverse effects of aberrant ISG expression. This regulatory mechanism may be important to maintaining physiological homeostasis. As reported previously, the negative regulatory phenomenon may provide us with a broader perspective for the helicase family proteins. However, the biological relevance is not clear yet since it is observed *in vitro* with the high exogenous introduction of DDX6 and no *in vivo* data is available. Further studies are needed to investigate the biological implications of the dual functionalities of DDX6 both *in vitro* and *in vivo* and the molecular mechanisms that mediate the signaling pathways.

The roles of DDX6 are multi-faceted in addition to regulating type I IFN response. DDX6 suppresses the differentiation program by degrading the translation of self-renewal and differentiation mRNAs to maintain adult progenitor cell function (Wang et al., 2015). DDX6 is also required for the promotion of *ATG16L1* during nitrogen starvation in HEK-293A cells (Liu et al., 2019). In *S. cerevisiae*, DDX6 restrains the expression of some ATG genes (Hu et al., 2015). The above study provided a hypothesis that DDX6 may regulate autophagy-related genes and influence autophagy processes. It has been reported that autophagy plays a role in promoting or inhibiting viral replication during viral infection (Fu et al., 2015; Choi et al., 2018). Furthermore, it remains to be explored whether DDX6 can regulate autophagy pathways during RNA virus infection. DDX6 also participates in the life cycle regulation of specific RNA viruses, including translation of viral RNA, replication, and viral capsid assembly (Ostareck et al., 2014). For example, the reduction of DDX6 is thought to alleviate HIV-1 translational inhibition (Nathans et al., 2009). For the dengue virus (DENV), DDX6 is involved in the replication and translation process by combining the 3 ‘UTR and 5’ UTR of DENV (Ward et al., 2011).

In addition to DDX6, other members of the DExD/H helicase family play significant roles in the antiviral response, which links mRNP function to RIG-I signaling. For example, DHX36 facilitates RIG-I signaling by inducing stress granule formation to resist RNA viruses (Yoo et al., 2014). DDX25 negatively regulated RIG-I signaling pathway and blocked IFN β production to facilitate DENV infection (Feng et al., 2017). DDX60 is a novel antiviral RNA helicase that facilitates RIG-I-like receptor-mediated signaling and is important for recognizing VSV, poliovirus, HSV-1, and SeV (Miyashita et al., 2011). More recently, it has been reported that DDX1 interacts with the FDMV 3D protein and inhibits virus replication (Xue et al., 2019). Many studies have shown that DEAD-box helicases are essential for RNA biogenesis. However, the details of their roles in these pathways have yet to be fully elucidated. Ultimately, a better understanding of the virus-host interactions could guide the antiviral therapies.

## Data Availability Statement 

The raw data supporting the conclusions of this article will be made available by the authors, without undue reservation.

## Author Contributions

RZ and ZW contributed to conception and design of the study. MY and MC organized the database. BL and DC performed the statistical analysis. RZ wrote the first draft of the manuscript. MC, MY, BL, YW, and ZW wrote sections of the manuscript. All authors contributed to the article and approved the submitted version.

## Funding

This work was supported by National Science Foundation of China (NSFC) (No. 31970149), The Major Research and Development Project (2018ZX10301406), Nanjing University-Ningxia University Collaborative Project (Grant# 2017BN04)

## Conflict of Interest

The authors declare that the research was conducted in the absence of any commercial or financial relationships that could be construed as a potential conflict of interest.

## Publisher’s Note

All claims expressed in this article are solely those of the authors and do not necessarily represent those of their affiliated organizations, or those of the publisher, the editors and the reviewers. Any product that may be evaluated in this article, or claim that may be made by its manufacturer, is not guaranteed or endorsed by the publisher.
