# Corrigendum: DEAD-Box Helicase DDX6 Facilitated RIG-I-Mediated Type-I Interferon Response to EV71 Infection

**DOI:** 10.3389/fcimb.2021.789723

**Published:** 2021-10-22

**Authors:** Rui Zhang, Min Cheng, Bingxin Liu, Meng Yuan, Deyan Chen, Yujiong Wang, Zhiwei Wu

**Affiliations:** ^1^ Center for Public Health Research, Medical School, Nanjing University, Nanjing, China; ^2^ State Key Lab of Analytical Chemistry for Life Science, Nanjing University, Nanjing, China; ^3^ Medical School and Jiangsu Key Laboratory of Molecular Medicine, Nanjing University, Nanjing, China; ^4^ School of Life Sciences, Ningxia University, Yinchuan, China

**Keywords:** EV71, DDX6, RIG-I, innate immune response, 2A^pro^

In the original article, there was an error. The conserved amino acid sequence of DEAD (Glu-Asp-Ala-Glu) in the first sentence of the abstract should be (Asp-Glu-Ala-Asp).

A correction has been made to the first sentence of the abstract section of our paper.

Corrected sentence: “Previous studies have shown that DEAD (Asp-Glu-Ala-Asp)-box RNA helicases play important roles in viral infection, either as cytosolic sensors of pathogenic molecules or as essential host factors against viral infection”.

The authors apologize for this error and state that this does not change the scientific conclusions of the article in any way. The original article has been updated.

## Publisher’s Note

All claims expressed in this article are solely those of the authors and do not necessarily represent those of their affiliated organizations, or those of the publisher, the editors and the reviewers. Any product that may be evaluated in this article, or claim that may be made by its manufacturer, is not guaranteed or endorsed by the publisher.

