# Supplementary material for: DEAD-Box Helicase DDX6 Facilitated RIG-I-Mediated Type-I Interferon Response to EV71 Infection
Source: Front Cell Infect Microbiol. 2021 Aug 13;11:725392. doi: 10.3389/fcimb.2021.725392 (PMC8414799; doi:10.3389/fcimb.2021.725392)
Supplement: Supplementary file 1 [file DataSheet_1.pdf]

Supplementary Material

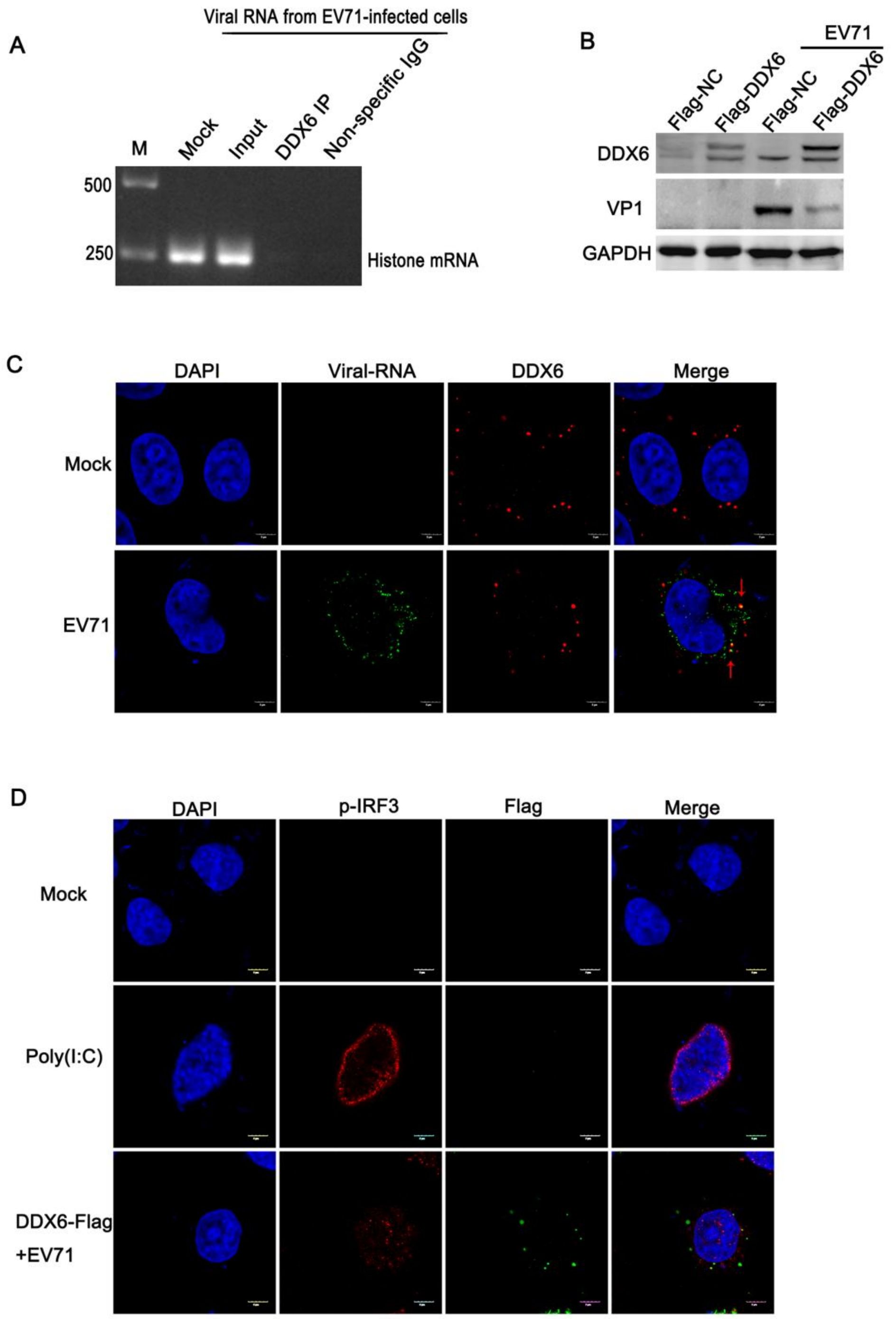

Figure 1

(A): RIP experiment with histone mRNA, which does not bind DDX6. (B): HEK 293 cells were transfected with a plasmid expressing DDX6 (pcDNA3.1-Flag-DDX6) or a control plasmid (pcDNA3.1-Flag). After 24 hours of transfection, the cells were treated with EV71 (MOI=2). 24 h post-infection, the cell lysates were analyzed by

western blot using antibodies specific for DDX6, VP1, and GAPDH. (C): HeLa grown in 12-well plates were infected with EV71 for 12 h or mock treatment. The cells were fixed and stained with antibodies against DDX6 (red), viral RNA (green) and DAPI (blue); the images were acquired using a confocal microscope. (D): HeLa with different treatments were fixed and stained with antibodies against p-IRF3 (red), Flag (green); the images were acquired using a confocal microscope.
